# Dynamic changes of nutrient composition throughout the entire life cycle of black soldier fly

**DOI:** 10.1371/journal.pone.0182601

**Published:** 2017-08-10

**Authors:** Xiu Liu, Xuan Chen, Hui Wang, Qinqin Yang, Kashif ur Rehman, Wu Li, Minmin Cai, Qing Li, Lorenzo Mazza, Jibin Zhang, Ziniu Yu, Longyu Zheng

**Affiliations:** 1 State Key Laboratory of Agricultural Microbiology, National Engineering Research Center of Microbial Pesticides, College of Life Science and Technology, Huazhong Agricultural University, Wuhan, China; 2 Livestock and Dairy Development Department, Poultry Research Institute Rawalpindi, Punjab, Pakistan; 3 College of Science, Huazhong Agricultural University, Wuhan, China; 4 University of Messina, Department of Chemical, Biological, Pharmaceutical and Environmental Sciences, Messina, Italy; Universita degli Studi della Basilicata, ITALY

## Abstract

Black soldier fly (BSF) larvae, *Hermetia illucens* L., develops on organic wastes, reducing ecological pollution and converting waste biomass into protein and fat rich insect biomass. BSF can replace increasingly expensive protein sources used in poultry, aquaculture and livestock compound diet formulation, such as fish meal and soybean meal, which holds the potential to alleviate future food and feed insecurity. The fate of nutritional spectra in BSF during its life cycle phases is still poorly understood. This study assessed metabolic changes in nutrition composition of BSF from egg to adult. A rapid increase of crude fat content was observed since the development of 4–14 days of larvae with its maximum level reaching 28.4% in dry mass, whereas the crude protein displayed a continuous decreasing trend in the same development phases with minimum level of 38% at larval phase (12 days) and peak level of 46.2% at early pupa stage. A sharp drop in crude fat was noticed from early prepupae to late pupae (24.2%, 8.2% respectively). However crude protein shows its maximum value being 57.6% at postmortem adult stage with 21.6% fat level. In addition, fatty acids, amino acids, minerals and vitamins composition in different development stages of BSF were presented and compared. Findings from this study could provide podium to food and feed industry for framing a strategy for specific molecular nutritional component intake into the diets of humans, aquaculture and animals. It is also indicated that BSF is a possible insect which can be applied to combating the food scarcity of countries where micronutrient deficiency is prevalent. Moreover it contributes to advance exploring for developmental and metabolic biology of this edible insect.

## Introduction

Due to rapid growth of world population, which is predicted to reach 9 billion people in 2050, the global food demand will rise by nearly 100% from 2005 to 2050[1,2], whereas animal feed and human food production through agriculture is expected to increase by 60%[3]. Future shortage for maize, rice, wheat and soybean was estimated approximately 67%, 42%, 38%, and 55% respectively[4]. The undernourished population increased by approximately 805 million in the developing countries which could lead to greater risk of local, national and global diseases outbreaks[3]. Therefore, the search for new food and feed sources contain high amount of protein and essential amino acids, fatty acids and micronutrients (calcium, iron, and zinc) becomes urgent.

As novel alternative protein resources for human food and animal feed, edible insects are globally appealing[5]. Edible insects are popular groups of organisms all over the world with potential food source, efficient food conversion rate, short period of breeding, and high protein content[6,7]. They are considered to be the major and potential animal protein source for its development and their utilization will create remarkable contribution to solution to the problems, such as protein resource shortage, confronted by food and feed industry[8,9]. According to literature[8], more than 2000 species of edible insects have been documented around the globe. The data revealed that the nutritional quality of edible insects was enough to fight against human malnutrition[10]. Insects contain large quantities of crude protein (CP), crude fat (CF) with high economic value are used to replace traditional protein sources such as fish meal, and soybean meal in the animal, poultry and aquaculture compound feed manufacturing industry[1,11].

*Hermetia illucens* (Diptera: Stratiomyidae), commonly known as black soldier fly, is distributed worldwide prominently in the equatorial tropics[12]. Its natural distribution includes Asia, Europe and southeastern United States, etc.[13–15]. The black soldier fly larvae (BSFL) are voracious consumers of organic wastes, including decaying fruits, vegetable waste, animal manure, and municipal organic waste[16–18].

Life cycle of the BSF can be divided into four phases: egg, larva, pupa, and adult stage[16,19]. During the special last period of larval stage, the prepupae migrates to the dry and suitable pupation site and converts into pupa[20]. The adult flies are neither pest nor disease vector. They survive on the fat stored in their larval stage, later on, they feed on nothing except only water[21,22]. The female BSF oviposit only around the edges of the larval food source, rather than on the food[23], therefore they do not transmit pathogens from the wastes[24]. Not only does this insect have the potential to reduce plant and animal waste pollution up to 50%, it also reduces the harmful bacteria and housefly population[1,5]. BSFL recycles wasted nutrition by converting poultry manure, swine manure, dairy manure[25], human feces[24], vegetable waste[26], and human food waste[6,27]. The manures and wastes provide BSF with approximately 40% protein and 30% fat[1]. Lipid accumulation varies based on various feeding materials[28]. BSF is widely used as excellent feed source in the animal and aquaculture feed industry[1,29–31]. As a novel biological raw material, BSFL can also be used for biofuel production[32–34], making Waste-to-Energy technology more practicable.

Most of previous researches on BSF have focused on the immature and adult life traits, basic biological developmental features, raising approaches, animal and plant waste treatment process[25,35,36], and the application of BSF to the animal and fish feed[1,37,38]. Moreover, prepupa was the stage frequently reported for nutrition composition analysis in previous studies. But no scientific data have been reported showing prepupa is better or more suitable to be used as feed ingredient than other forms of BSF. To best of authors knowledge no former reports on the nutritional composition variations during various life stages of BSF were available except for the data of the mature larval and prepupal stage[29].

Therefore, exploration of the fluctuations in the nutrients such as minerals, vitamins, amino acids (AAs), fatty acids (FAs), CP and CF level during whole life cycle of BSF is essential. This study mainly focused on uncovering variations of nutrients in different life phases of BSF from egg to adult, especially in the most important feeding larval phase, so as to provide theoretical basis for the exploitation of various BSF products and other edible insects.

## Materials and methods

### Source of flies

BSF used in this study was Wuhan strain[25]. Wuhan strain laboratory colony was maintained in a greenhouse at the National Engineering Research Center of Microbial Pesticides, Huazhong Agricultural University (HZAU), Wuhan, Hubei, China, for about 9 years.

### Feed

To keep consistency, commercial broiler chicken feed (520) purchased from Charoen Pokphand Group, Wuhan China, was used and provided to BSF for development in the present research. Water content was measured by taking 10 g samples of chicken feed and then placing them in three glass containers, and the material was dried at 60°C for dry matter. The CP, CF, calcium, total phosphorus and crude ash were analysed according to the procedures described in determination of nutritional index section, whereas the values of sodium chloride, crude fiber, and methionine and cysteine were adopted as provided by manufacturer. The nutritional composition of this chicken feed was listed in Table 1. Before being fed to the larvae, chicken feed was moistened with tape water to get proper water content (60% in present study).

**Table 1:** Nutrient composition of chicken feed used in current study (%).

Moisture	Crude protein	Calcium	Total phosphorus	Crude ash	Sodium chloride	Crude fiber	Methionine and cystine
**7.8±1.1**	21.0±0.2	1.1±0.2	0.5±0.0	7.9±0.1	0.6±0.3	5.9±0.1	0.9

Values are in Mean ± S.E.

### Experimental design and operation

The experiment was carried out at the greenhouse of the State Key Laboratory of Agricultural Microbiology, HZAU, Wuhan, Hubei, China. The experiment was done in triplicates. About 1300 egg mass (mostly one egg mass is contributed from one gravid female) were collected within a 5 h gap and used for this part of experiment. Firstly, eggs were divided equally (440 egg mass each incubator) and placed in three incubators, allowing to hatch 26°C, relative humidity (RH) 65% - 70%. Egg hatching was monitored every 8 h and resulted larvae were removed timely. To obtain possible synchronization in BSF developmental stages, the neonate larvae hatched within a 12 h from all of the three incubators were selected to be used for current investigation. Moreover, hatched larvae were separated equally by weight into three groups as replicates to achieve a same larval density. Furthermore larvae were placed into three individual plastic basins (height 31 cm, diameter 11 cm) with 500 g of moist chicken feed having 60% water content. When the volume of larvae and feed reached three-quarters of the volume of each basin, the same group of larvae from one basin were numerically equally divided into two groups, and transferred into two larger plastic containers (height 62 cm, diameter 15 cm). Larval rearing containers were covered with gauze to reduce drying of the medium. During the whole process of cultivation, same quantity of 500 g to 2500 g of moist chicken feed was added each day to each container as needed, depending on the rate of digestion of the larvae. Feeding process of experimental animal was stopped when about 50% of the larvae reached prepupal stage.

### Sampling procedure

#### Egg sampling

Fresh eggs of BSF were collected from colony cages using blocks of corrugated cardboard (3 cm × 5 cm) placed on the wall of plastic containers with moist artificial diet served as oviposition attractant. To get enough egg samples for analysis of nutritional component, egg collecting started at 9:00 am stopped at 5:00 pm on each experimental day, and then egg-containing corrugated cardboards were dissected to remove all the egg mass out. Eggs were then weighed and stored at -20°C before freeze-drying and nutritional analysis could be performed.

#### Pre-feeding neonate larvae sampling

Eggs were allowed to hatch in the incubators, at 26°C, with RH 65% - 70% as described in experimental design and operation. The neonate larvae sampling were collected in equal weight from each replicate. The resulted neonate larvae from this procedure were weighed 100 g in total and stored at -20°C, until adequate sample obtained for nutritional analysis. No food supplied to the neonate larvae (pre-feeding).

#### Feeding stages dynamic sampling

The larvae reared on chicken feed and after feeding for 4 days, first random sampling was conducted and then at 6, 7, 9, 12 and 14 days of feeding from each replicate; and 100 g of larvae were sampled each time from each replicate (both containers). The larval samples were washed, air dried, weighed, and conserved at -20°C. The feeding stage sampling was stop as 50% appearance of prepua (14 days) as described in experimental design and operation section.

#### Post-feeding stages dynamic sampling

Newly emerged prepupae were removed from the experimental containers timely every day. Prepupae from the 2^nd^ day after prepupation started were selected and used for further post-feeding dynamic sampling. The 100 g of prepupae from the 2^nd^ day were stored at -20°C and defined as early prepupa (E-prepupa). Pupation at 26°C, RH 65% - 70% was monitored every 12 h for each replicate. Once pupation was observed, pupae were immediately removed. Freshly emerged pupa and the remaining prepupae left in containers were respectively defined as early pupa (E-pupa) and late prepupa (L-prepupa). After sampling of E-pupa, pupae from each replicates were placed into cages (65 cm × 115 cm) and allowed to be monitored for adult emergence. Late pupa (L-pupa) sample were defined and collected once adult emerged. After two days of emergence, adult male and female flies were sampled from experimental cages and separately placed in ziplock bags and stored at -20°C.

Experimental dynamic sampling was carried out from mid-November 2015 to late-December 2015. All samples were frozen at -20°C and dried using vacuum freeze drier (FD-1A-50, Beijing Boyikang Laboratory Instruments Co. Ltd., China).

### Determination of nutritional index

Prior to the analysis the eggs, pre-feeding, feeding and post feeding samples of BSF from various life cycle phases were freeze-dried until constant weight. While freeze drying may result in less complete moisture removal compared to the oven drying, this difference is minimal and freeze drying guarantees a better preservation of nutrients. Nutritional components determination of freeze dried BSF samples from this study was completed by PONY TEST Co., China, with reference to China National Standards (GB).

#### Crude protein

Kjeldahl method was used for crude protein (CP) determination of BSF samples. Briefly, protein in the sample was disintegrated under the condition of catalytic heating, released ammonia then reacted with sulfuric acid resulting in ammonium sulfate. The alkaline distillation was applied to free ammonia which was subsequently absorbed by boric acid and further titrated with hydrochloric acid titrant. CP content was calculated according to the acid consumption multiplied by the conversion factor (GB 5009.5–2010).

#### Crude fat

The crude fat (CF) was estimated by taking 2 g from larval sample. It was put into the 50 ml of test tube, next 8 ml of water and 10 ml of hydrochloric acid were added to the tube. The tube was placed in 70–80°C water bath until the sample was digested completely. The digested sample was added of 10 ml ethanol following transferred to 100 ml of mixing cylinder with stopper. The 5 ml of petroleum ether-ether mixture was added into the cylinder and allowed to stand. After taking supernatant out the cylinder, the remaining liquid was transferred to the flask, and the flask was placed on a water bath and repeatedly evaporated to constant weight. The quantity after eliminating the solvent was the total lipid content (GB/T 5009.6–2003).

#### Ash

The BSF sample (2 g) was put into crucible, and fully carbonized on the hot plate until smoke-free, then the sample was placed in a muffle furnace and burned at 550 ± 25°C for 0.5 h until the ash was formed, and ash content was calculated by weighing (GB 5009.4–2010).

#### Fatty acids composition

The fatty acids (FAs) spectra of BSF life stages were analyzed by taking 0.5 g sample. The sample was put into 50 ml of flask and 6 ml of sodium hydroxide methanol solution was added. The sample was saponified and then 7 ml of boron trifluoride was added. After extraction for 3 minutes, a methyl ester solution was obtained. The 0.2 μl of methyl ester solution was taken into the syringe and subjected to gas chromatography analysis to determine the FAs composition (GB/T 17376–2008).

#### Amino acids profile

The amino acids (AAs) profile was analyzed according to methods described by GB/T 5009.124–2003. Briefly, 0.5 g of sample was put into hydrolysis tube, added 10 ml of hydrochloric acid (6 mol/l), and test tube was sealed with nitrogen. The sealed hydrolysis tube was placed in electro-thermal constant temperature dry box at 110 ± 1°C and was hydrolyzed for 22 h, cooled, dried, and was used automatic amino-acid analyzer after being dissolved by 1 ml of buffer with pH 2.2.

#### Vitamin E estimation

The vitamins in the BSF life cycle phases was determined by taking 10 g of sample into 250 ml of Erlenmeyer flask and dissolved with 50 ml of water. Then 100 ml of the vitamin C ethanol solution was added to the Erlenmeyer flask, the mixture was mixed, and 25 ml of potassium hydroxide aqueous solution was added to saponify. The saponified solution was transferred to 500 ml of separatory funnel; 100 ml of petroleum ether was added to the separatory funnel after extraction, then extract liquor transferred to the test tube. Then the vitamin E content was determined by liquid chromatography (GB 5413.9–2010).

#### Elemental analysis

The mineral elements phosphorus, potassium, sodium, zinc, calcium and iron in the dry mass (DM) of BSF larval meal were determined as following the mentioned procedure with reference to China National Standards (GB).

Phosphorus: 0.1 g of sample was put into 100 ml of Kjeldahl flask. The sulfuric acid and perchloric acid-nitric acid was successively added (3 ml of each) to the Kjeldahl flask. The flask was placed on the digestion furnace until after the reaction was completed. The mixture was transferred to a 100 ml of volumetric flask. The 2 ml of molybdic acid, 1 ml of sodium sulfite solution, and 1 ml of hydroquinone solution were added into the test tube. Then leave it for 30 min, the liquid absorption was measured with a spectrophotometer at the wavelength of 660 nm (GB/T 5009.87–2003).

Potassium and sodium: 0.5 g of sample was placed in 250 ml of beaker, 20 ml of mixed acid was added to the beaker for digestion and the beaker was heated on a hot plate. After the sample was digested completely, the digestive juice was transferred to the 10 ml of scale tube with water, fixed to the scale. The potassium was estimated by introducing digested sample into a flame photometer and the emission intensity was measured at wavelength 766.5 nm. Similarly sodium was determined; when digested sample was introduced into the flame photometer, emission intensity was measured at wavelength 589 nm (GB/T 5009.91–2003).

Zinc: 0.5 g of sample was placed in a porcelain crucible, which was burned by a minor fire until smokeless. The carbonized samples were transferred to a horse-fired furnace and ashed for about 8 h at 500±25°C. Then 10 ml of hydrochloric acid was added to the porcelain crucible, the residue was dissolved and moved into a 50 ml of volumetric flask. The treated sample solution was introduced into atomic absorption spectrophotometer at wavelength 213.8 nm (GB/T 5009.14–2003).

Calcium: 0.5 g of sample was placed in 250 ml of beaker. The 20 ml of mixed acid was added to the beaker for digestion and the beaker was heated. After the sample was digested completely, the digestive juice was transferred to the 10 ml of scale tube with Lanthanum oxide (20 g/l), fixed to the scale. The digested sample was introduced into atomic absorption spectrophotometer and the emission intensity was measured (measurement conditions: wavelength 422.7 nm) (GB/T 5009.92–2003).

Iron: 0.5 g of sample was placed in 250 ml of beaker, and 20 ml of mixed acid was added to the beaker for digestion and the beaker was heated. After the sample was digested completely, the digestive juice was transferred to the 10 ml of scale tube with water, fixed to the scale. The digested sample was introduced into atomic absorption spectrophotometer and the emission intensity was measured (measurement conditions: wavelength 248.3 nm) (GB/T 5009.90–2003).

### Statistical analysis

The statistical analysis was performed by SPSS 16.0 (SPSS Inc., Chicago, IL, USA). The results of the CP, CF and ash in DM of BSF life cycle were analyzed by one-way analysis of variance (ANOVA), followed by Tukey’s HSD (honestly significant differences) for post-hoc testing to compare the significance (P) between the means of different life cycle stages. P<0.05 was considered to indicate a significant difference between the values compared. The chicken feed composition and variations of CP, CF, AA, FA and DM content in the individual BSF life phases, and individual adult female and male were expressed as Mean ± SE.

## Results

**Fig 1:**
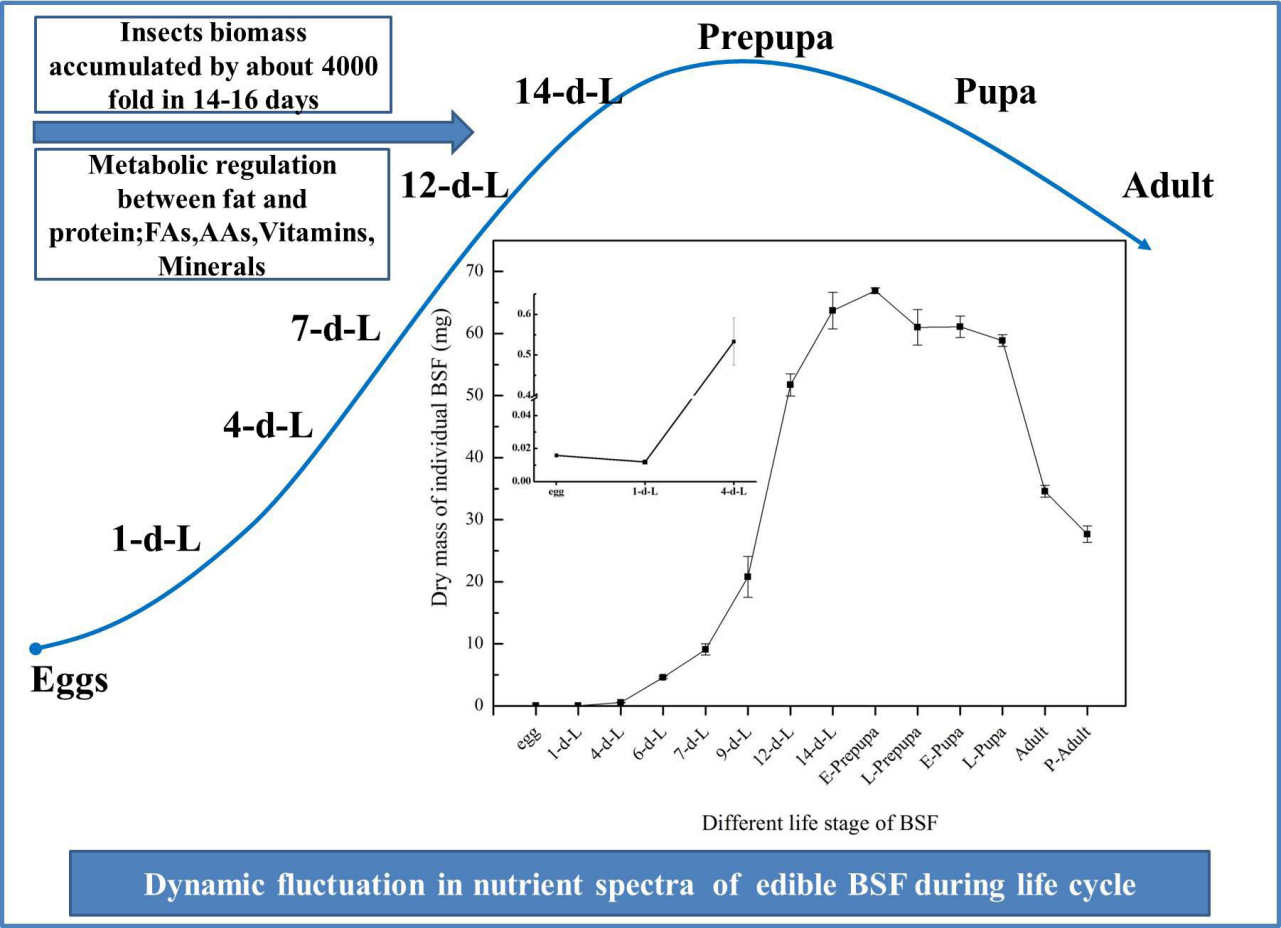
Changes in BSF nutritional spectra and biomass accumulation during the life cycle. All the results were calculated by dry mass. d-L: day-larvae.

This study presented the nutritional composition of BSF in different phases of life span. The variation in the nutritional spectra and larval biomass accumulation of BSF during different stages of life cycle is shown in Fig 1.

### Crude protein, crude fat, ash and dry mass composition of BSF

The results indicated that there were marked variations in the nutritional composition of BSF during different phases of life cycle (Table 2). The crude Protein relative content was 45% in eggs, whereas it increased to 56% in neonatal larvae after hatching for one day (Table 2). However, it gradually reduced to 38% on the 12^th^ day of larval development. But an upsurge of CP content was observed in the on 14^th^ day larvae (mature larvae) was 39.2%, and then followed by a gradual climb and stabilization in later phases of life cycle, such as E-prepupa (40.2%), L-prepupa (40.4%), E-pupa (46.2%), L-pupa (43.8%) and adults (43.8%, 44.0% in male and female respectively) (Table 2).

**Table 2:** Crude protein, crude fat and ash content of BSF in diverse life cycle steps.

Life cycle phases	Crude protein (%)	Crude fat (%)	Ash (%)
**Egg(<12h)**	45.0±0.12^e^	15.8±0.06^f^	4.0±0.15^gh^
**1-d-L**	56.2±0.06^b^	4.8±0.08^j^	5.0±0.17^g^
**4-d-L**	54.8±0.28^c^	5.8±0.26^j^	10.5±0.40^a^
**6-d-L**	54.2±0.15^c^	9.6±0.06^h^	10.0±0.06^abc^
**7-d-L**	46.0±0.21^d^	13.4±0.17^g^	9.2±0.25^bcde^
**9-d-L**	42.0±0.10^g^	22.2±0.28^e^	8.4±0.23^def^
**12-d-L**	38.0±0.35^j^	22.6±0.15^e^	7.8±0.41^f^
**14-d-L**	39.2±0.06^i^	28.4±0.06^c^	8.3±0.26^ef^
**E-prepupa**	40.2±0.15^h^	28.0±0.25^c^	8.8±0.21^cdef^
**L-prepupa**	40.4±0.21^h^	24.2±0.28^d^	9.6±0.06^abcd^
**E-pupa**	46.2±0.12^d^	8.2±0.12^i^	9.6±0.15^abcd^
**L-pupa**	43.8±0.21^f^	7.2±0.03^i^	10.2±0.32^ab^
**Female**	43.8±0.06^f^	30.6±0.26^b^	2.8±0.06^h^
**Male**	44.0±0.10^f^	32.2±0.42^a^	3.0±0.06^h^
**P-adult**	57.6±0.26^a^	21.6±0.36^e^	3.6±0.23^h^
**SBM[63]**	44.51	1.84	6.13
**FM[64]**	66.0	10.8	21.8

All the results were calculated by dry mass. N-d-L: n-day-larvae. E—prepupa: early prepupa. L—prepupa: late prepupa. E-pupa: early pupa. L-pupa: late pupa. P-adult: postmortem adult. Female and male sampling was complete in two days after emergence. The proportion of male and female in postmortem adult samples was about 1:1. Data are expressed as mean ± S.E; values within a column with different superscripts differ from each other at P<0.05.

Crude fat was about 15.8% in egg stage (Table 2). Then it felled to 4.8% after one day’s hatching, while different life stages indicated the divergent CF content (Table 2). Fat was dramatically accumulated through the whole feeding process with larval development; it rose from 4.8% on 1^st^ day of larval age to 28.4% on 14^th^ day (Table 2). And then it dropped to 8.2% and 7.2% in E-pupa and L-pupa respectively. After metamorphosis and emerged into adults, CF content bounced back to normal level, 30.6% for female and 32.2% for male.

In contrast with the fluctuation of CP and CF content at different stages, is valuable from Table 2 that the ash content of BSF samples remained relatively stable from 4^th^ day of larvae to L-pupa, ranging from 7.8% to 10.3%. While ash content in egg and adult samples were significantly lower. Male BSF has higher level of CP, CF and ash content than the female in DM; moreover, CP content of postmortem adult (P-adult) samples (57.6%) was surprisingly higher than that of other pre-feeding, feeding and post-feeding stages.

**Fig 2:**
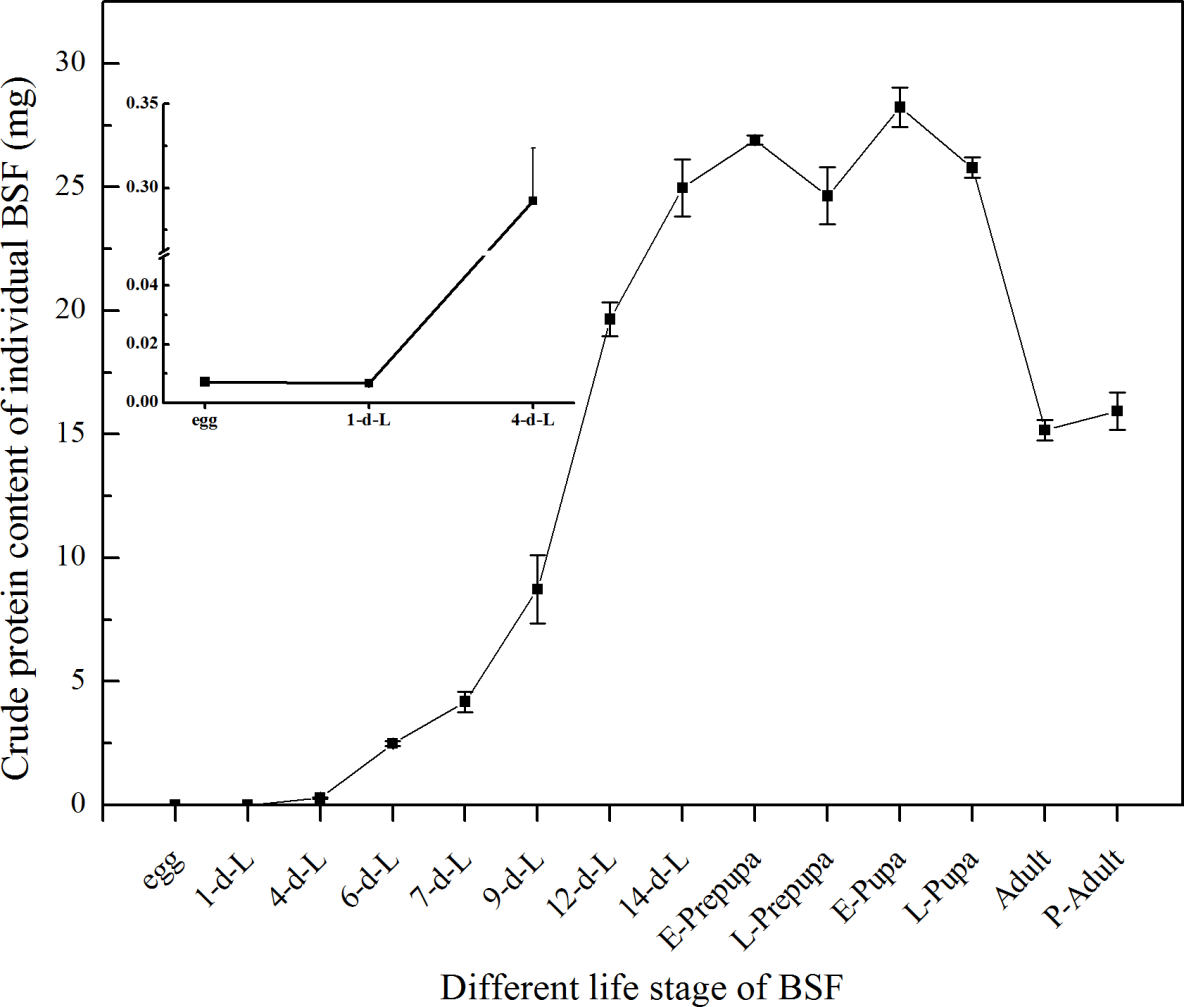
Variations of crude protein content in individual BSF dry mass during life phases. All the results were calculated by dry mass. d-L: day-larvae. E-prepupa: early prepupa. L-prepupa: late prepupa. E-pupa: early pupa. L-pupa: late pupa. P-adult: postmortem adult. Female and male sampling was complete in two days after emergence. The proportion of male and female in postmortem adult samples was about 1:1. Bars indicate the standard error of the means (n = 3).

The variations of CP absolute content of the individual BSF at different life phases were shown in Fig 2. Individual CP absolute content rose gradually from 0.006 mg (1^st^ day larvae) in the larval stage to its peak level of 28.228 mg in E-pupa (Fig 2). A sharp increase was detected since the 4^th^ day of larval development. Then, it was followed by a decrease in L-pupa (25.798 mg) and P-adult (15.149 mg). The analysis of the CP absolute content in larval stage indicated that the highest level of CP emerged respectively in mature larval phase at 14^th^ days of development (24.974 mg) and E-prepupa (26.935 mg).

**Fig 3:**
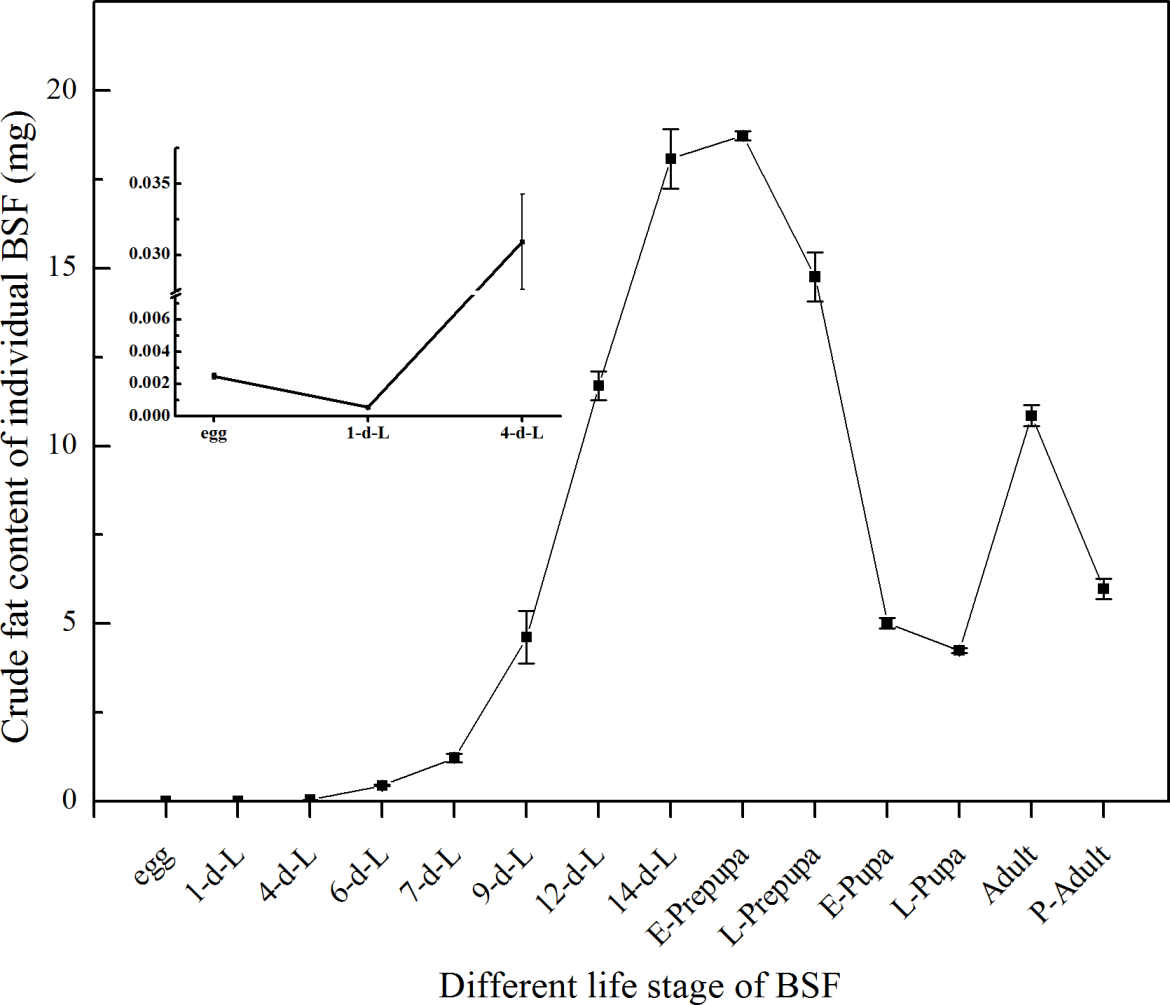
Modification of crude fat in dry mass in individual BSF life cycle. All the results were calculated by dry mass. d-L: day-larvae. E-prepupa: early prepupa. L-prepupa: late prepupa. E-pupa: early pupa. L-pupa: late pupa. P-adult: postmortem adult. Female and male sampling was complete in two days after emergence. The proportion of male and female in postmortem adult samples was about 1:1. Bars indicate the standard error of the means (n = 3).

Crude Fat absolute content of individual BSF at different life stages were shown in Fig 3. Individual CF absolute content increased in neonatal larvae at initial hatching stage. It reached at highest level in the mature larvae stage (18.091 mg) and in E-prepupa stage (18.760 mg), respectively. The following stages witnessed a decline of CF absolute content, including in L-prepupa (14.786 mg), E-pupa (5.010 mg) and L-pupa (4.241 mg). Same as the relative content, CF absolute content in adult increased again back to a decent level (10.854 mg) which is essential for reproduction energy demand and decreased in P-adult (5.681 mg) after death.

**Fig 4:**
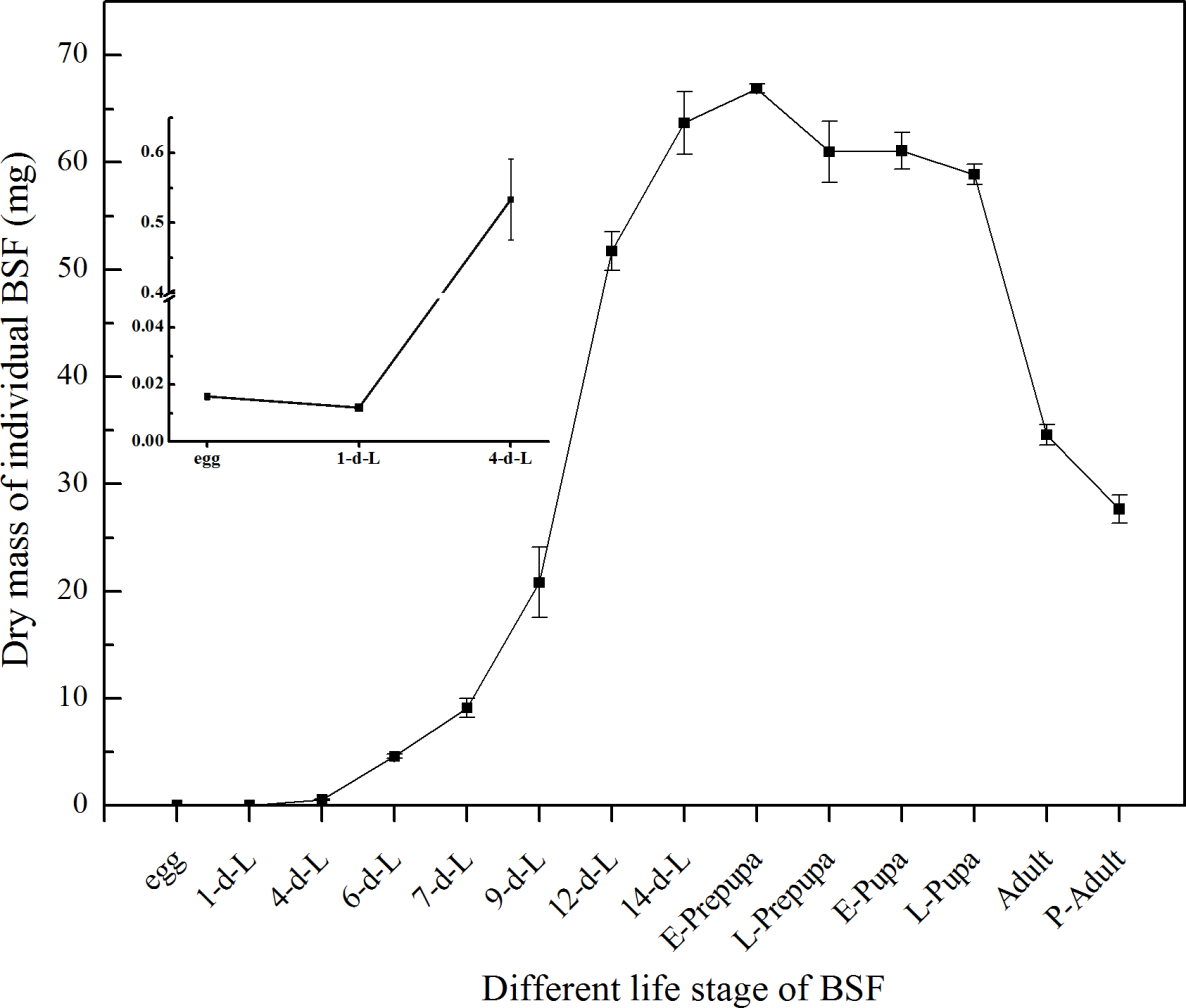
Dry mass fluctuation of individual BSF in different life stages. All the results were calculated by dry mass. d-L: day-larvae. E-prepupa: early prepupa. L-prepupa: late prepupa. E-pupa: early pupa. L-pupa: late pupa. P-adult: postmortem adult. Female and male sampling was complete in two days after emergence. The proportion of male and female in postmortem adult samples was about 1:1. Bars indicate the standard error of the means (n = 3).

**Fig 5:**
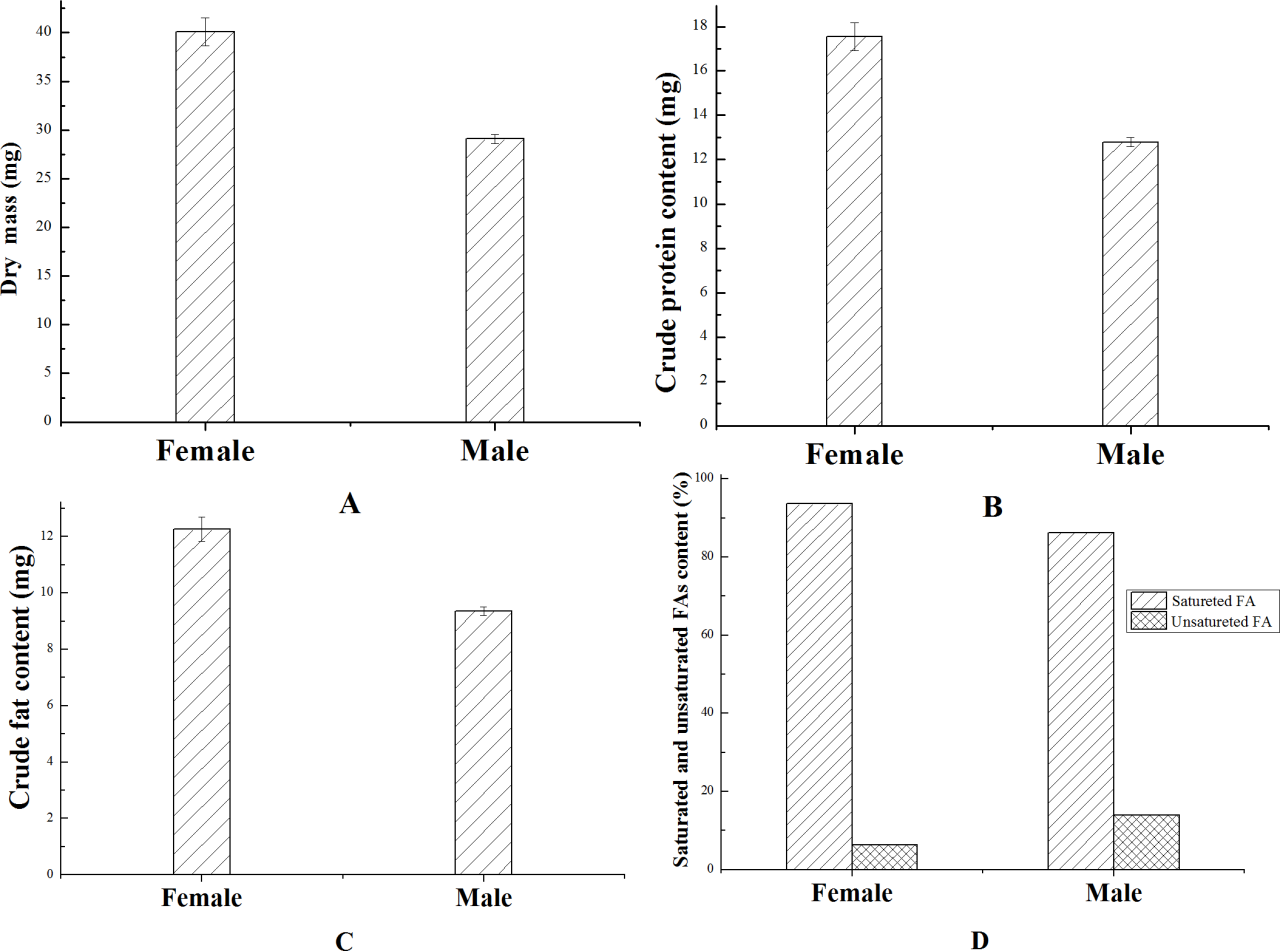
Nutritional spectra in dry mass of female and male adult BSF. (A) show dry mass variation, (B) illustrate crude protein content alteration, (C) demonstrate crude fat content modification, (D) explains saturated and unsaturated fatty acids changes. (Female and male sampling in two days after emergence). Bars indicate the standard error of the means (n = 3).

It was perceived that larvae experienced the rapid accumulation of biomass since the 4^th^ to 6^th^ day of larval stage, whereas the variation in the dry biomass development during the life cycle of BSF was shown in Fig 4. It was noted that the DM of individual BSFL was increases by about 4000 folds from 14 to 16 days of development (Figs 1 and 4). In addition, female adult BSF had a higher level of DM, CP, CF and FAs absolute contents than male adult (Fig 5).

### Variations of amino acids profile during BSF life span

The nutritional profile of BSF protein was evaluated by examining the AAs composition through different development phases, and results were presented in Table 3. It is indicated that a variety of essential AAs were synthesized in every growth stage of BSF from egg to adult. For example, the content of lysine which usually considered being a limiting amino acid in plant-protein was relatively abundant throughout the lifespan (19.0–29.8 g/kg). Moreover, this study demonstrated that mostly the highest level of AAs contents emerged in the early development of larvae (4^th^ - 6^th^ day) that followed by a continuous decrease until the maturation of larva, and that AAs contents level remained stable in late stages, such as E-prepupa, L-prepupa, E-pupa and L-pupa (Table 3). Interestingly, content of a large portion of AAs were remarkably higher in adult stage than other life stages in DM (g/kg).

**Table 3:** Amino acid compositions of BSF in different life history traits and comparison with soybean meal and fish meal.

	Egg(<12h)	1-d-L	4-d-L	6-d-L	7-d-L	9-d-L	12-d-L	14-d-L	E-Prepupa	L-prepupa	E-pupa	L-pupa	Female	Male	P-adult	SBM[63,65]		FM[64,66]
**Asparagine(g/kg)**	41.6±0.75^b^	39.2±0.15^ef^	40.9±0.24^bc^	34.8±0.04	33.2±0.15^h^	29.6 ±0.24^i^	32.4±0.20^h^	36.3±0.12^g^	35.8±0.15^g^	35.1±0.11^g^	35.8±0.10^g^	32.5 ±0.15^h^	39.8±0.13^cd^	32.8±0.05^h^	48.8±0.71^a^		32	37
**Threonine(g/kg)**	20.0±0.13^bcd^	22.8±0.18^b^	23.2±2.88^a^	21.7±0.16^bc^	20±0.09^bcd^	17.8±0.10^cd^	17.1±0.15^e^	18.4±0.11^cd^	18.5±0.15^cd^	18.1±0.05^cd^	18.6±0.09^cd^	17.2±0.06^e^	20.7±0.10^bcd^	18.4±0.08^cd^	23.8±0.06^b^		18	29
**Serine(g/kg)**	21.5±0.08^b^	23.1±0.07^a^	21.6±0.08^b^	20.2±0.04^d^	17.4±0.12^e^	14.4±0.09^j^	15.8±0.13^i^	16±0.15^hi^	16.2±0.10^ghi^	16.5±0.10^fg^	16.9±0.05^f^	16.4±0.08^gh^	15.8±0.06^i^	16.2±0.05^ghi^	20.8±0.08^c^		18	19
**Glutamic acid(g/kg)**	55.8±0.20^e^	65.4±0.95^b^	69.4±0.19^a^	64.4±0.15^c^	59±0.11^d^	49.1±0.20^f^	43.7±0.08^h^	45.2±0.07^g^	42.0±0.11^i^	38.4±0.07^j^	38.2±0.05^j^	34.2±0.20^k^	46.6±0.18^g^	49.3±0.15^f^	59±0.07^d^		18	27
**Proline (g/kg)**	21.8±0.08^gh^	29.1±0.06^b^	30.6±0.14^a^	29.4±0.52^b^	27.6±0.12^c^	23.6±0.24^e^	22.8±0.09^ef^	21.9±0.16^fgh^	21.8±0.16^gh^	21.6±0.09^h^	22±0.11^fgh^	21.8±0.21^gh^	22.6±0.14^fg^	21.6±0.15^h^	26.4±0.08^d^		16	—
**Glycine(g/kg)**	17.0±0.17^fg^	26.6±0.24^a^	24.4±0.05^b^	24.8±0.18^b^	22.1±0.14^d^	19.2±0.18^e^	17.6±0.04^f^	17.8±0.18^f^	20.0±0.21^e^	21.8±0.14^d^	23.2±0.22^c^	24.3±0.13^b^	17.2±0.19^fg^	16.6±0.11^h^	22.2±0.16^d^		37	9
**Alanine(g/kg)**	22.0±0.24^j^	34.2±0.09^b^	34±0.04^b^	43.6±0.12^b^	33.2±0.18^c^	30.2±0.08^d^	25.1±0.04^f^	23.3±0.13^h^	22.5±0.18^ij^	22.8±0.23^hi^	23.0±0.16^hi^	23.2±0.08^hi^	24.3±0.07^g^	27.4±0.18^e^	36.0±0.13^a^		30	36
**Valine(g/kg)**	19.0±0.08^de^	24.3±0.13^a^	24.7±0.11^a^	24.2±0.09^a^	21.2±0.16^b^	19.2±0.19^cde^	18.2±0.14^d^	18.7±0.21^d^	20.4±0.75^bc^	19.2±0.12^cde^	20.2±0.14^bcd^	20.0±0.27^bcd^	20.3±0.09^bc^	18.8±0.18^a^	24.3±0.12^a^		41	31
**Methionine(g/kg)**	8.4±0.06^h^	23.8±0.16^e^	19.4±0.14^g^	22.1±0.17^f^	22.6±0.12^f^	18.8±0.15^g^	22.4±0.13^f^	22.4±0.16^f^	26.8±0.14^d^	31.4±0.18^c^	33.6±0.54^b^	40.6±0.13a	18.4±0.17^g^	21.6±0.19^f^	33.4±0.11^b^		32	16
**Isoleucine(g/kg)**	16.8±0.08^ef^	21.4±0.19^a^	20.0±0.23^b^	20.4±0.17^b^	17.7±0.21^cd^	16.4±0.13^efg^	14.8±0.15^h^	15.6±0.18^gh^	16.6±0.10^ef^	16.0±0.14^fg^	16.9±0.16^de^	16.0±0.08^fg^	18.3±0.11^c^	17.2±0.13^de^	21.8±0.33^a^		46	26
**Leucine(g/kg)**	30.8±0.22^d^	36.8±0.13^a^	34.0±0.09^c^	35.0±0.11^b^	29.8±0.16^e^	28.2±0.08^g^	25.4±0.09^i^	27.1±0.14^h^	28.0±0.13^g^	28.0±0.09^g^	29.2±0.18^ef^	28.0±0.08g	31.4±0.31^d^	29.0±0.08^f^	37.4±0.13^a^		46	33
**Tyrosine(g/kg)**	20.2±0.32^hi^	23.4±0.14^e^	25.7±0.13^cd^	19.4±0.11^i^	21.0±0.11^gh^	20.2±0.18^hi^	22.4±0.21^f^	25.5±0.19^d^	28.4±0.17^a^	26.8±0.15^b^	26.6±0.11^bc^	25.8±0.08^cd^	22.6±0.17^ef^	21.7±0.23f^g^	26.2±0.17^bcd^		12	19
**Phenylalanine(g/kg)**	17.0±0.08^g^	18.2±0.16^ef^	21.0±0.42^b^	18.7±0.12^cde^	17.3±0.05^fg^	17.3±0.09^fg^	17.0±0.12^g^	18.6±0.12^de^	19.0±0.08^cde^	19.3±0.13^cd^	19.6±0.17^c^	17.4±0.21^fg^	21.0±0.12^b^	17.2±0.18^g^	22.0±0.21^a^		23	26
**Lysine(g/kg)**	23.8±0.14^f^	29.0±0.54^bc^	29.8±0.28^b^	28.4±0.14^c^	24.1±0.17^f^	21.4±0.19^g^	21.0±0.14^g^	23.2±0.22^f^	23.1±0.15^f^	21.4±0.12^g^	21.6±0.19^g^	19.0±0.09^h^	27.2±0.17^d^	25.7±0.14^e^	31.6±0.16^a^		28	48
**Histidine(g/kg)**	38.5±0.56^f^	53.2±0.39^b^	49.2±0.19^c^	55.0±0.48^a^	46.9±0.26^d^	41.0±0.12^e^	34.6±0.19^jk^	31.6±0.25^l^	34.9±0.14^ijk^	36.6±0.11^gh^	33.4±0.23^k^	36.4±0.17^ghi^	37.6±0.19^fg^	35.3±0.28^hij^	52.9±0.58^b^		25	14
**Arginine(g/kg)**	26.0±0.12^c^	30.3±0.21^a^	28.5±0.13^b^	20.4±0.14^ef^	19.5±0.13^fg^	17.0±0.33^j^	18.0±0.08^ij^	20.5±0.14^ef^	21.2±0.53^e^	20.4±0.18^ef^	21.2±0.17^e^	19.0±0.16^gh^	26.0±0.14^c^	23.7±0.11^d^	30.0±0.44^a^		62	34

All the results were calculated by dry mass. N-d-L: n-day-larvae. E—prepupa: early prepupa. L—prepupa: late prepupa. E-pupa: early pupa. L-pupa: late pupa. P-adult: postmortem adult. Female and male sampling was complete in two days after emergence. The proportion of male and female in postmortem adult samples was about 1:1. Data are expressed as mean ± S.E; values within a row with different superscripts differ from each other at P<0.05.

### Variations of fatty acids composition during BSF lifecycle

**Fig 6:**
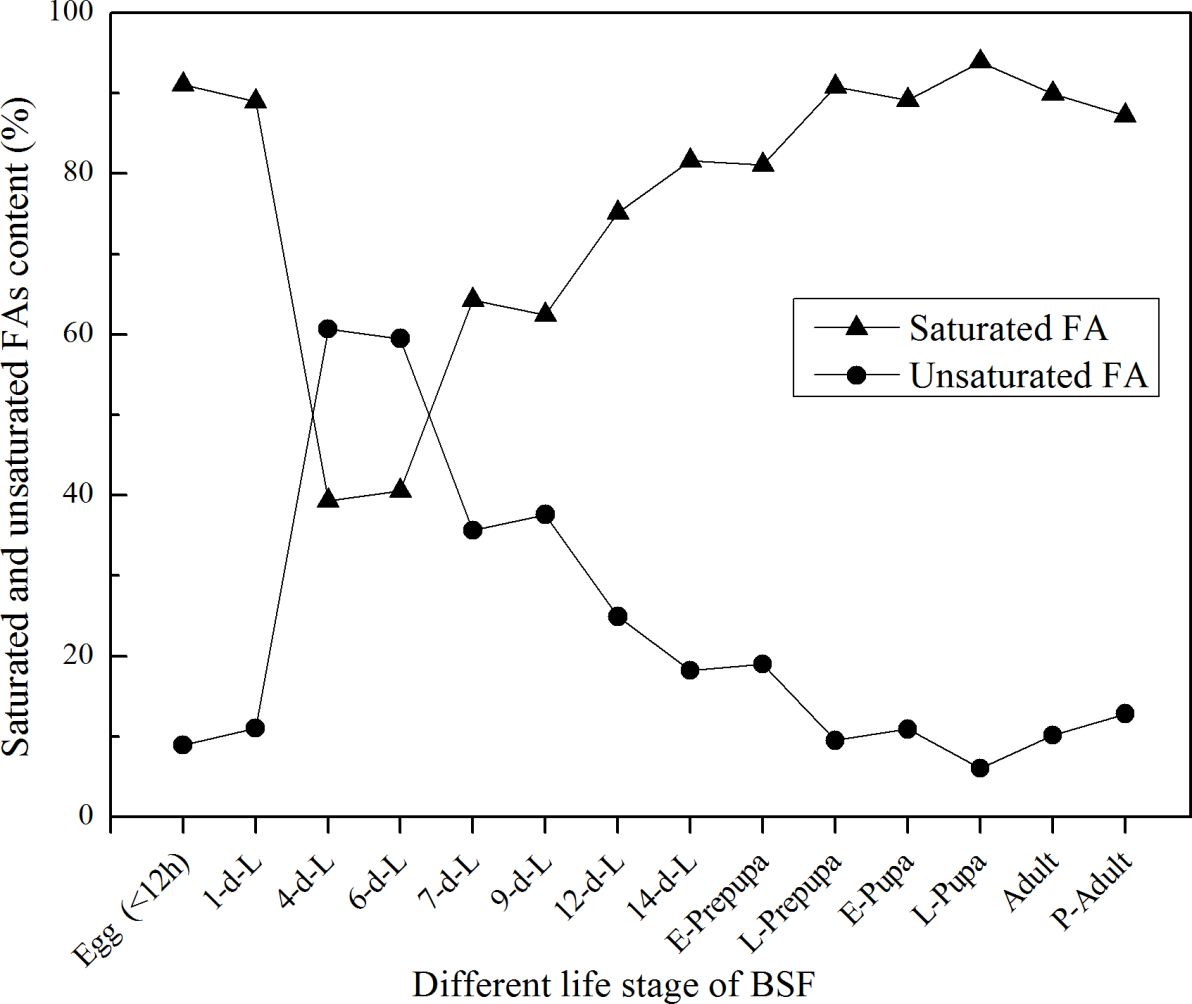
Alteration of saturated and unsaturated fatty acids in the dry mass of individual BSF during metamorphosis. All the results were calculated by dry mass. d-L: day-larvae. E-prepupa: early prepupa. L-prepupa: late prepupa. E-pupa: early pupa. L-pupa: late pupa. P-adult: postmortem adult. Female and male sampling was complete in two days after emergence. The proportion of male and female in postmortem adult samples was about 1:1.

Fatty acids contents in different life stages of BSF were shown in Table 4, which indicated that the FAs composition varied considerably from one stage to another. BSF fed on chicken feed in this study showed satisfactory levels of essential FAs (Table 4). Linoleic acid (18:2, ω-6) and α-linolenic acid (18:3, ω-3) respectively reached their maximum levels, namely, 31.4% and 1.6% on 6^th^ day of larval development. Oleic acid (18:1) peaked on 4^th^ day of larval development accounting for about 36.4% of BSFL CF, and then dropped up to the adult. Synthetic quantity of Lauric acid (12:0) was consistently high through the entire life cycle of BSF excluding 4-day-old larvae, accounted for 16%~70% of the total fat. Nevertheless, the 4-day-old larvae contained maximum types of FAs. Whereas the contents of unsaturated FAs were reached at peak level as shown in Fig 6. The BSF in early larval stage contained many unsaturated FAs. However, the contents of unsaturated FAs reduced gradually after its prepupal stage (Fig 6).

**Table 4:** Fatty acids compositions of BSF in different life steps (calculated by fat percentage).

	Egg (<12h)		1-d-L	4-d-L	6-d-L	7-d-L	9-d-L	12-d-L	14-d-L	E-prepupa	L-prepupa	E-pupa	L-pupa	Female	Male	P-adult
**Caproic 6:0 (%)**	—		—	0.6±0.02^a^	—	—	—	—	0.1±0.03^b^	—	—	—	—	—	—	—
**Decanoic 10:0 (%)**		1.3±0.06^bcd^	—	0.2±0.02^h^	0.3±0.02^gh^	0.4±0.02^g^	0.6±0.02^f^	0.9±0.03d^e^	1.1±0.01^c^	1±0.03^cd^	0.9±0.02^cde^	0.9±0.03^de^	0.8±0.02^de^	1±0.04^cd^	1±0.02^cd^	1.6±0.06^a^
**Lauric 12:0 (%)**		70.6±0.49^bc^	71.8±0.54^bc^	7.6±0.11^j^	16.4±0.18^i^	34±0.21h	38.2±0.17^g^	53.9±0.21^f^	61.4±0.09^e^	62.5±0.28^e^	73.4±0.65^b^	72±0.14^bc^	78.4±0.57^a^	78.9±0.62^a^	70.2±0.15^cd^	69.8±0.19^d^
**Myristic 14:0 (%)**		5.2±0.11^e^	4.9±0.07^ef^	2.6±0.05^g^	4.6±0.06^f^	8.6±0.09^d^	8.7±0.06^d^	10±0.13^a^	10.2±0.09^a^	9.4±0.12^b^	10.4±0.11^a^	9.2±0.08^bc^	8.8±0.11^cd^	9.4±0.05^b^	9.2±0.02^bc^	10.4±0.08^a^
**Myristoleic 14:1 (%)**		—	—	—	—	0.3±0.03^a^	—	0.3±0.02^a^	0.2±0.01^a^	—	—	—	—	—	0.2±0.02^a^	—
**Pentadecanoic 15:0 (%)**		—	—	0.1±0.01	—	—	—	—	—	—	—	—	—	—	—	—
**Palmitic 16:0 (%)**		2.8±0.09^l^	2.4±0.05^l^	22.8±0.17^a^	14.6±0.08^c^	18±0.11^b^	12.2±0.07^d^	8.8±0.0.08^e^	7.8±0.05^f^	7.2±0.05^g^	5.5±0.04^i^	6.3±0.06^h^	5.3±0.04^i^	3.9±0.05^k^	5.2±0.06^ij^	4.8±0.05^j^
**Palmitoleic 16:1 (%)**		1±0.02^g^	—	0.7±0.01^h^	2.3±0.02^d^	3.8±0.02^a^	3.9±0.03^a^	3.2±0.04^b^	2.5±0.05^c^	1.8±0.05^e^	1±0.04^g^	1±0.04^g^	0.6±0.01^h^	0.7±0.03^h^	1.6±0.02^f^	2.2±0.03^d^
**Heptadecanoic 17:0 (%)**		—	—	0.2±0.01	—	—	—	—	—	—	—	—	—	—	—	—
**Stearic 18:0 (%)**		2.1±0.06^e^	2.3±0.06^e^	4.8±0.07^a^	3.8±0.05^b^	3.2±0.05^c^	2.6±0.06^d^	1.4±0.04^f^	1±0.03^g^	1±0.04^g^	0.6±0.01^hi^	0.7±0.02^h^	0.6±0.01^hi^	0.4±0.08^i^	0.6±0.03^hi^	0.6±0.01^hi^
**Elaidic 18:1 (%)**		—	—	0.1±0.01^a^	—	0.1±0.01^a^	0.1±0.01^a^	0.1±0.01^a^	0.1±0.02^a^	—	—	—	—	—	—	—
**Oleic 18:1 (%)**		3.4±0.03^g^	6.1±0.13^f^	36.4±0.26^a^	24.2±0.55^a^	15.8±0.14^b^	16±0.17^b^	10.4±0.09^d^	7.8±0.06^e^	7±0.06^ef^	3.4±0.05^g^	3.7±0.04^g^	2±0.04^h^	2±0.05^h^	4.2±0.09^g^	3.5±0.06^g^
**Linoleic 18:2 (%)**		4.5±0.18^ij^	5±0.12^hi^	22.4±0.46^a^	31.4±0.53^a^	14.8±0.10^d^	16.6±0.11^c^	10.3±0.09^e^	7.2±0.13^f^	9.6±0.09^e^	4.8±0.05^hi^	5.8±0.08^gh^	3.2±0.05^j^	3.5±0.07^ij^	7.5±0.13^f^	6.8±0.08^fg^
**α-Linolenic 18:3 (%)**		—	—	0.9±0.02^b^	1.6±0.15^a^	0.8±0.02^bc^	1±0.05^b^	0.6±0.02^cd^	0.4±0.02^de^	0.6±0.02^cd^	0.3±0.02^e^	0.4±0.02^de^	0.2±0.01^e^	0.2±0.00^e^	0.4±0.05^de^	0.3±0.02^e^
**Arachidic 20:0 (%)**		0.5±0.02^a^	—	0.3±0.02^b^	0.3±0.02^b^	0.1±0.01^c^	0.1±0.01^c^	0.1±0.01^c^	—	—	—	—	—	—	—	—
**Eicosenoic 20:1 (%)**		—	—	0.1±0.01	—	—	—	—	—	—	—	—	—	—	—	—
**Docosanoic 22:0 (%)**		2.2±0.11^a^	1.2±0.05^b^	0.1±0.01^c^	0.2±0.03^c^	—	—	—	—	—	—	—	—	—	—	—
**Lignoceic 24:0 (%)**		6.4±0.14^a^	6.4±0.09^a^	—	0.3±0.02^b^	—	—	—	—	—	—	—	—	—	—	—
**Osenic 24:1 (%)**		—	—	0.1±0.01	—	—	—	—	—	—	—	—	—	—	—	—

All the results were calculated by dry mass. N-d-L: n-day-larvae. E—prepupa: early prepupa. L—prepupa: late prepupa. E-pupa: early pupa. L pupa: late pupa. P-adult: postmortem adult. Female and male sampling was complete in two days after emergence. The proportion of male and female in postmortem adult samples was about 1:1. Data are expressed as Mean ± S.E; values within a row with different superscripts differ from each other at P<0.05.

### Vitamin and minerals contents in potential commercial stages of BSF

Table 5 displays the vitamin and mineral contents in mature larvae at 14^th^ day and E-prepupa with uniform nutritional parameters. The mature larvae and E-prepupa phase of BSF both were rich in vitamin E. Vitamin E content in the larval stage (6.68 mg/100g) was higher than that in E-prepupa stage (3.26 mg/100g). Moreover, BSF contained numerous minerals calcium, phosphorus, sodium, iron and zinc (Table 5). The content of calcium reached up to 2900 mg/100g in mature larvae, while the content of calcium in the DM was significantly higher in E-prepupa stage (3000 mg/100g) than in mature larval (14^th^ day) stage (2900 mg/100g). Similar results were found for phosphorus. Phosphorus content in E-prepupa stage (620 mg/100g) almost doubled that in mature larvae (350 mg/100g) and the proportion of calcium to phosphorus was 4.84 ~ 8.28: 1 approximately. In addition, the contents levels of sodium, iron and zinc were higher in mature larval phase (100 mg/100g, 200 mg/100g, and 61.40 mg/100g, respectively) than in the E-prepupa stage (50.10 mg/100g, 7.49 mg/100g and 3.27 mg/100g, respectively).

**Table 5:** Vitamin and mineral content of *Hermetia illucens*.

Vitamin and mineral	Mature larvae (14^th^ day)	E-prepupa
**Vitamin E (mg/100g)**	6.7±0.64	3.3±0.42
**Calcium (mg/100g)**	2900.0±13.57	3000.0±18.45
**Phosphorus (mg/100g)**	350.0±6.11	620.0±9.85
**Sodium (mg/100g)**	100.0±2.48	50.1±1.41
**Iron (mg/100g)**	200.0±4.27	7.5±0.95
**Zinc (mg/100g)**	61.4±1.71	3.3±0.29

All the results were calculated by dry mass. Data are expressed as Mean ± S.E.

## Discussion

Black soldier fly has been considered as a potential environmentally sustainable nutritious alternative to conventional livestock, poultry, and aquaculture[1,39,40]. Previous experimental studies reported the fluctuation of nutritional ingredients of BSF by providing various feed sources for larval development[6,29,41,42], and mainly focused on the last larval and prepupal stage[29]. However this study has provided an insight into nutritional composition amendments and variation in BSF life phases from egg to adult. Informational evidence obtained is supportive for insect farmer, aquaculture farmer, pet owner, researchers, and for the whole feed manufacturing industry of livestock, poultry, and aquaculture. Moreover investigation findings are valuable to improve the mass breeding and product development system of this mini-livestock, so as to meet future challenge of supplying secure proteins for approximately 9 billion people expected to dwell on planet by 2050, which is likely to be the major priority for the global community[3,43].

In this study, CP accounted for about 39.2% at larval phase, and then rose up to 40.2% in prepupal stage. The current findings are consistent with those of previous research on BSF fed on chicken feed, which reported that CP ranged from 34% to 42% in larval phase and from 31% to 46.2% in prepupal phase respectively[44–46]. While other research reported 42% in larval stage and 38% prepupal state of BSF fed on unknown feed[29]. About 43.2% and 41% of CP was reported in prepupal stage of BSF grown on swine manure and poultry manure respectively[47], and 38% - 46.3% of CP in larval stage of BSF grown on byproduct derived from food[6].It is previously reported that the level of protein content in BSF depended on feed types[6] and feeding rate[44]. Therefore it is certain that CP content in BSF varies with diverse feed sources that they feed on.

Fat content in BSF was also reported previously to vary with diet types. For example, in prepupal meal, 15% - 34.8% of CF was reported noted by[1], 34% of CF was reported when BSF grew on beef cattle and poultry manure, 28% in swine manure[47,48], and 42% - 49% on oil rich feed source[49]. Fat content in BSF was enhanced by applying different feed supplement. Microbes have shown great potential facilitating BSF to better dispose wastes and co-convert waste nutrients into insect biomass, which could ultimately boost the biodiesel and defatted larval meal production, making waste-to-energy more realistic[28,34,50]. This study found that CF content accounted for 28% in mature larval and early prepupal stage which was in accordance with the results of previous research on the nutritional composition in BSF. The comparison of increasingly expensive traditional protein source used in the poultry and aquaculture compound feed; soybean meal (SBM) and fishmeal (FM)[1,37] with the BSF meal was presented in Table 1.

What is more notable is the fluctuation trend of larval DM, CP, and CF contents over time during BSF development. The intense rise in the dry larval biomass was recorded from 1^st^ day to 4^th^ day of larval development (Fig 1). The DM accumulation continues to rise gradually up-to 9^th^ day, then a strong rise was noted that continue at E-prepupa stage. Whereas the DM was after the mature larval (14^th^ day) and E-prepupa stage continue to decline and reach its lowest in adult stage of BSF. It was recorded that the DM of individual BSFL was increases by about 4000 folds in 14 to 16 days of development (Figs 1 and 4); similarly CP and CF absolute content increased rapidly and reached the highest level in the mature larvae and E-prepupa (Figs 2 and 3). Crude protein content increased just after hatching, and then it gradually reduced and remained relatively stable (approximately 40%) from mature larval stage (14^th^ day) to L-prepupa, while it noticeably increased in pupal and adult stages. We speculate that there may be a comprehensive regulation between protein and fat metabolism and metabolic flux shifted more into fat synthesis during larval growth, storing adequate energy for metamorphosis and reproduction. As for the fluctuation trend of CF, this study found that it firstly dropped from 15.8% to 4% after hatching into neonate larvae, indicating that egg shell left may contain more fat, crucial for eggs to prevent moisture loss. CF content gradually increased in the larval phase and reached the highest level of 28% in mature larval (14^th^ day) and prepupal phases, and then it experienced a significant reduction in pupal stages. Remarkable decrease of fat content during pupation may be due to fat body dissociation and energy cost for metamorphosis, which have been reported for other insects[51,52]. Adult fat body could be remodeled, leading to a fat content sudden increased in BSF adults essential for adults to complete mating and oviposition[53]. Moreover, absolute CP content was documented 26.89 mg in E-prepupa and 28.23 mg in E-pupae while CF in E-prepupa and E-pupae period was 18.73 mg and 5.01 mg of DM respectively, higher than that in individual BSF. These variations in the nutrient composition of CP and CF was previously reported in larval stage and prepupal stage[29,44].

The AAs composition reported in this study (Table 3) is consistent with that reported in previous studies in larval stage[47], prepupal stage[41,46], both larval and prepupal stages of BSF[29]. Some other studies also reported the variation in AAs level in BSF fed on different wastes (beef and swine manure)[47,54]. A more detailed determination of the change of essential and non-essential AAs contents in whole life stages of BSF and comparison with SBM and FM were conducted in present work (Table 3).

Regarding the FAs spectra of BSF fed on the chicken feed, as shown in Table 4, a low level of caproic acid (6:0) was observed in 4-day-old larvae. This finding is different from that of the previous study, which denied the presence of this fatty acid both in BSF and in other insect species, such as yellow meal worm and Argentinian cockroaches[6]. This investigation also reported the existence of osenic (24:1) in early larvae stage in BSF fed on chicken feed, while previous study denied its presence on the food waste groups[6]. BSF in every stage of its life cycle shows satisfactory high level of essential FAs, including linoleic (18:2), and α-linolenic acid (18:3). Despite BSF fed on chicken feed have lower level of α-linolenic acid in larval (0.4%) and prepupal (0.6%) stage than beef (2%) and pork (1.12%)[55], high level of α-linolenic acid was observed on 6^th^ day (1.6%) and 9^th^ day (1%) of development. In addition, linoleic content was determined to be about 7.2% and 9.6% in mature larval stage and prepupal stage respectively, and even a higher level on 6^th^, 7^th^, 9^th^ and 12^th^ days of development, which were much higher than that in beef (2.0%) and pork (0%) reported in other research[55,56]. These essential FAs act as material basis for the synthesis of long chain PUSFAs, such as arachidonic acid, eicosapentaenoic and docosahexaenoic acid. They are also necessary to maintain cell membrane, brain function and nerve impulse transmissions under normal conditions. These FAs also participate in such processes such as atmospheric oxygen transferring to plasma, hemoglobin synthesis, and cell division[57]. The PUSFAs from the ω-3 series (18: 3) and ω-6 (18: 2) may prevents cardiovascular diseases and cancers[56]. Fats in BSF have good application potential because a proper level of FAs are highly desirable to the human and animal diets[55,56].

Table 5 shows the contents level of vitamin, micro and macro minerals which are considered to be nutritionally important to animal and human diets. At this point, edible insects abundant with vitamin and minerals are likely to reduce the consumption of dairy, meat and animal protein products[5,58,59]. It was also found that vitamin E and most minerals were available in mature larvae and E-prepupa stages. Therefore the application of BSF larvae as feed additive in human feed may be protect against many heart diseases and cancers caused by reactive free radicals, as vitamin E is the major lipid soluble antioxidant in the cell antioxidant defense system[60]. It can be concluded that the high level of vitamin E in BSF can meet the demands of animals and humans[11,57].

The amount of mineral elements, such as calcium, phosphorus, sodium, iron and zinc were measured and listed in Table 5, both in mature larval and prepupal stage which are the most economic effectiveness commercial stages. A higher level of minerals in prepupal than in larval stage is also evaluable: the possible reason lies in the cuticle formation in prepupal period. But the levels of sodium, iron and zinc were higher in larval than in prepupal stage (Table 5). Mineral contents level is consistent with that appreciable in relevant studies of BSF’s prepupal stage[47,54]. Moreover, high level of mineral elements in BSF reported by our study will support the statement that edible insect could help to combat food shortage in those countries where micronutrient deficiencies are prevalent[61,62].

Facts of BSF nutritional components will be useful to meeting the demand of development of new food additives and to establishing a cost-effective and well-optimized mass insect rearing industry. Compared with other edible insects, BSF is richer in lipid, protein, vitamin E and minerals. Therefore, the whole body of this mini-livestock is a treasure worthy of our in-depth exploration.

## Conclusion

Insect usage in poultry, fish, and human diet as alternative green environmental friendly sustainable source of proteins and fats, has recently observed a surge. The knowledge of the BSF nutrient spectra and in particular, the bioavailability of protein, fats, AAs, FAs, vitamins and minerals, mainly the variation in different life cycle stages, is presently sparse. It was noted that the DM of individual BSFL was increases by about 4000 folds during 14 to 16 days of development. The CP and CF absolute content increased rapidly and reached the highest level in the E-prepupa. Therefore mature larvae and E-prepupa are the life cycle stages for generation of BSF meal.

It is the first systematic attempt to examine the nutrient components in BSF, presenting the fluctuation trend of nutritional components in almost every stage of its lifespan from egg to adult. Especially the metabolic patterns over time for development of protein and fats, two of the most important nutrients, were preliminarily revealed. To further validate this metabolic rule, future studies can be extended to explore and evaluate nutritional components of BSF fed on different waste materials during its various life stages. Findings in this study can promote better usage and provide basis for further exploring of developmental and metabolic biology of this resource insect.
